# Supplementary material for: Functional protein mining with conformal guarantees
Source: Nat Commun. 2025 Jan 2;16:85. doi: 10.1038/s41467-024-55676-y (PMC11695924; doi:10.1038/s41467-024-55676-y)
Supplement: Supplementary file 1 — Supplementary Information [file 41467_2024_55676_MOESM1_ESM.pdf]

Functional protein mining with conformal guarantees
Supplementary Information

Ron S. Boger[1,2,3], Seyone Chithrananda[2,4], Anastasios N. Angelopoulos[4,5], Peter H. Yoon[2,6], Michael I. Jordan[5,7], and Jennifer A. Doudna[2,3,6,8,9,10,11]

[1]Biophysics Graduate Group, University of California, Berkeley; Berkeley, CA, USA.
[2]Innovative Genomics Institute; University of California, Berkeley, CA, USA.
[3]California Institute for Quantitative Biosciences, University of California, Berkeley; Berkeley, CA, USA.
[4]Department of Electrical Engineering and Computer Sciences, University of California, Berkeley; Berkeley, CA, USA.
[5]Department of Statistics, University of California, Berkeley; Berkeley, CA, USA.
[6]Department of Molecular and Cell Biology, University of California, Berkeley; Berkeley, CA, USA.
[7]Howard Hughes Medical Institute, University of California, Berkeley; Berkeley, CA, USA.
[8]Molecular Biophysics and Integrated Bioimaging Division, Lawrence Berkeley National Laboratory; Berkeley, CA, USA.
[9]Department of Chemistry, University of California, Berkeley; Berkeley, CA, USA.
[10]Gladstone Institutes; San Francisco, CA, USA.
[11]Gladstone-UCSF Institute of Genomic Immunology; San Francisco, CA, USA.

December 13, 2024

1 Supplementary Figures

*Corresponding author: doudna@berkeley.edu

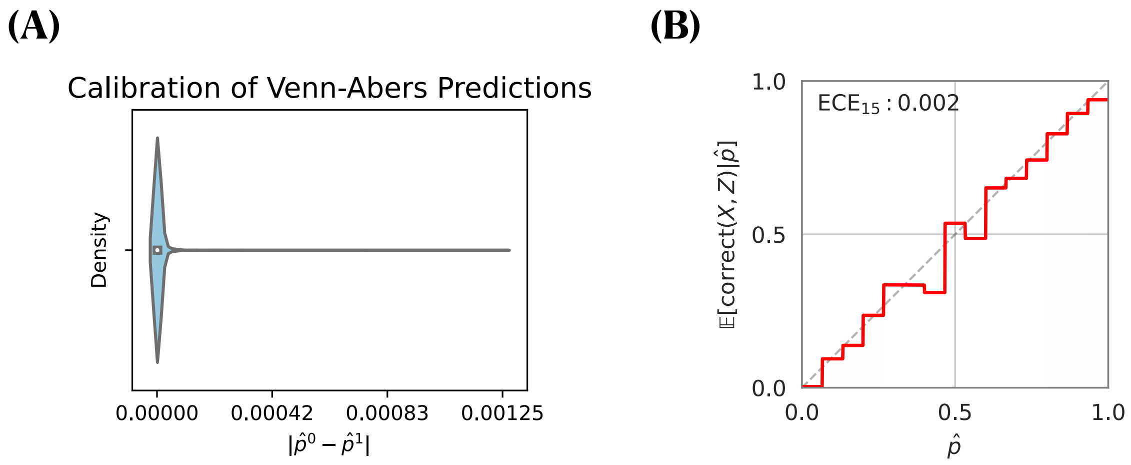

Supplementary Figure 1: Calibration and reliability of the Venn-Abers predictors, assigning probability from similarity score. **(a)** The difference between Venn-Abers $\hat{p}^0, \hat{p}^1$ is low, indicating that probability computations are well calibrated. **(b)** Venn-Abers predictor shows excellent reliability with Expected Calibration Error $ECE = .002$. The horizontal axis represents "predicted match frequency" and the vertical axis represents "true match frequency".

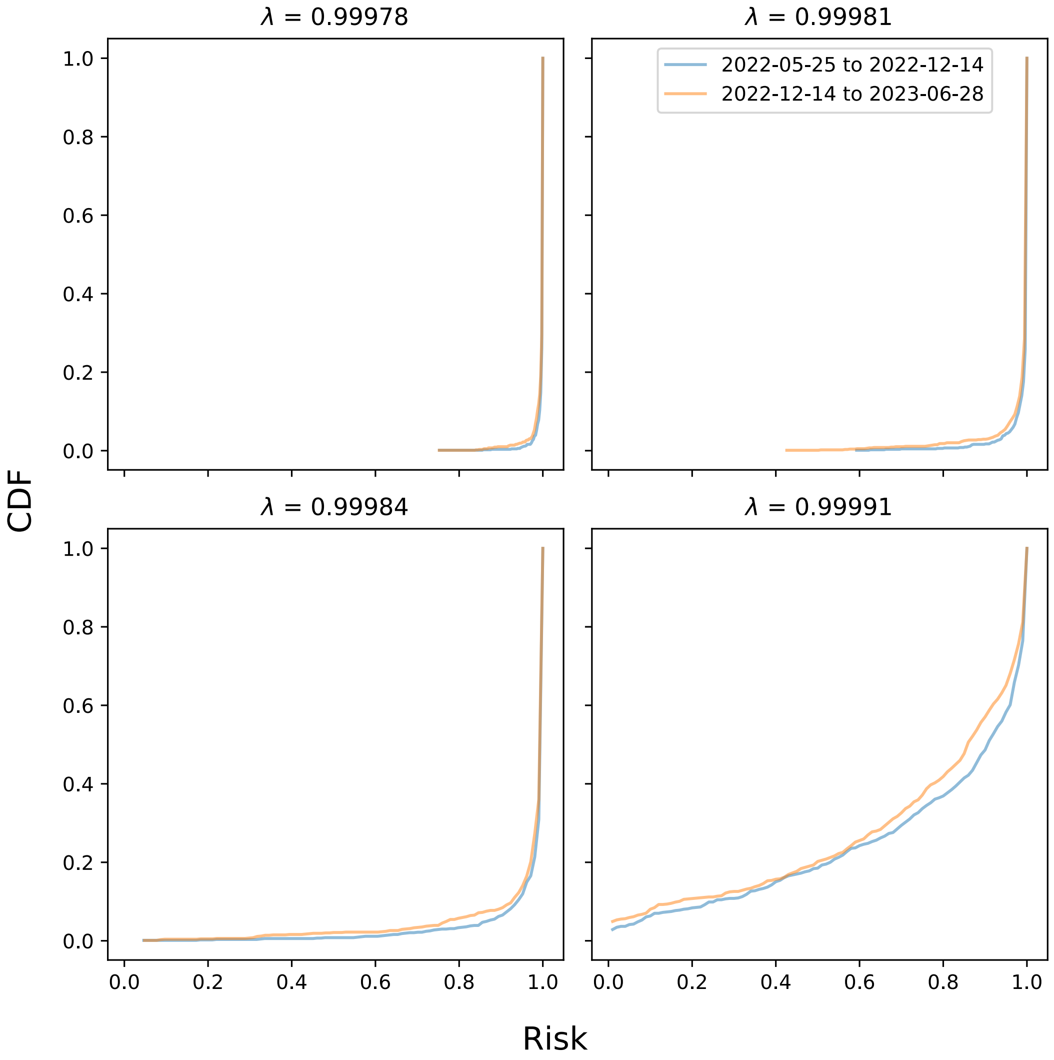

Supplementary Figure 2: We make plots of the empirical FDR loss at different λ thresholds corresponding to the $\{20, 50, 80, 99\}^{\mathrm{th}}$ percentile of similarity scores between unknown and known proteins. The unknown proteins are the test set, and the calibration set is composed of known proteins. The cumulative density function of the losses roughly overlap, indicating that the calibration and test sets are close to exchangeable as far as the risk function is concerned. Note that the calibration and test sets are split temporally, thus directly speaking to our ability to trust exchangeability on future, unknown proteins.

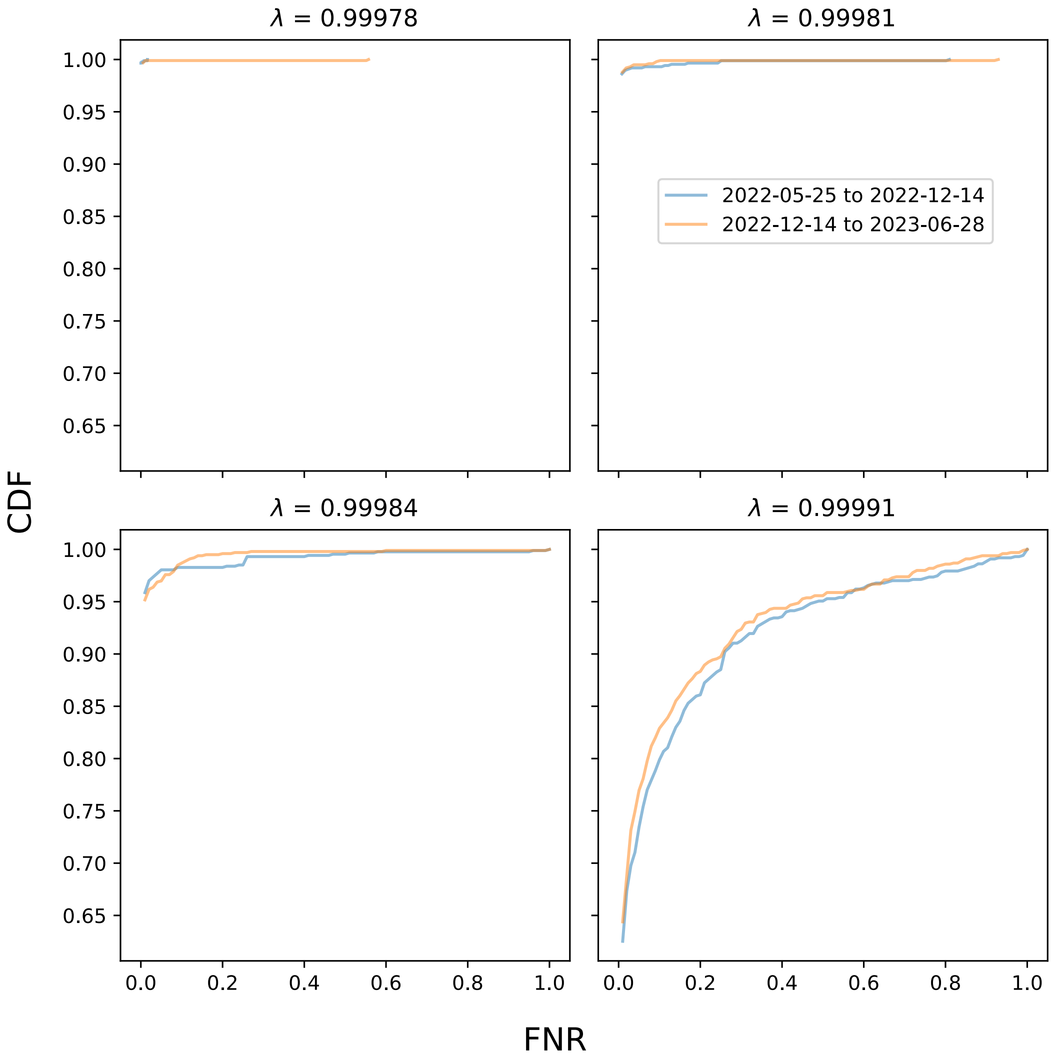

Supplementary Figure 3: We test to see the FNR loss is exchangeable at different λ thresholds corresponding to the $\{20, 50, 80, 99\}^{\text{th}}$ percentile of similarity scores between unknown and known proteins. The the cumulative density function of the losses roughly overlap, indicating exchangeability.

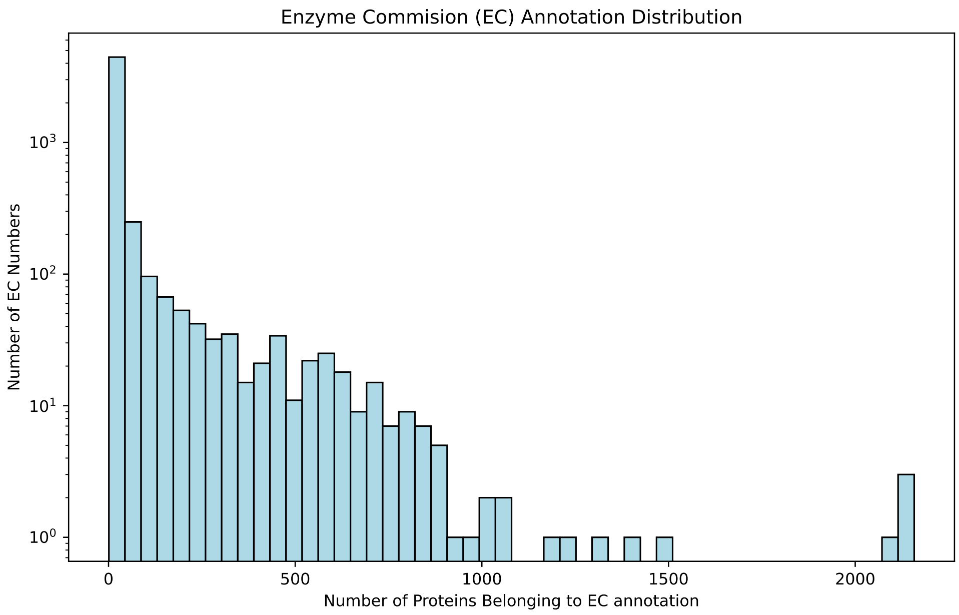

Supplementary Figure 4: Histogram of the EC annotation distribution within the CLEAN training set. 4498 of the 5242 EC annotations in CLEAN have less than 50 protein examples, illustrating the need for a calibration technique aware of the hierarchical nature of enzyme assignments.

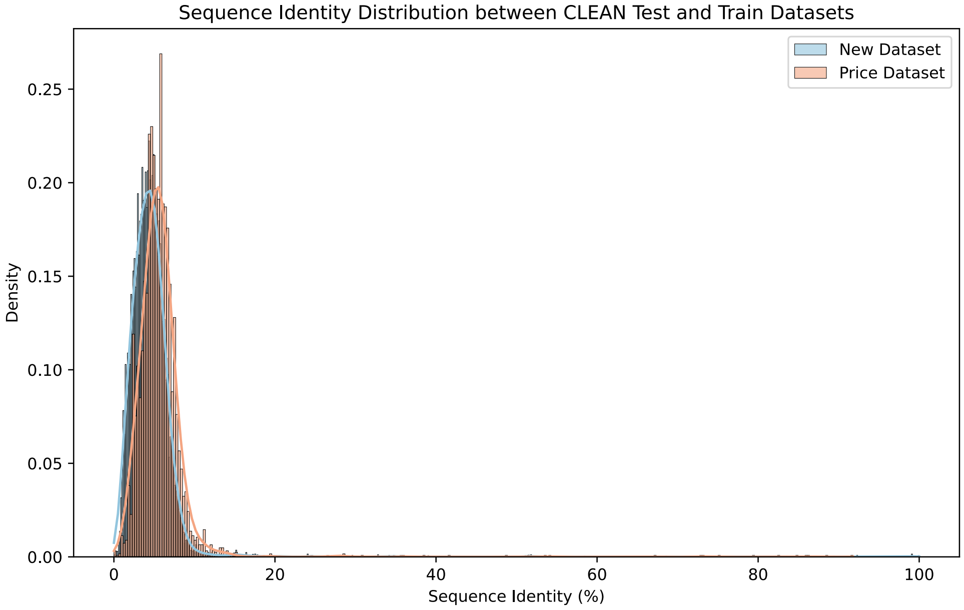

Supplementary Figure 5: Histogram of the sequence identity distribution for test enzymes in *New* and *Price* towards exact EC matches (serial number) in the CLEAN training set. Similar to the slight distribution shift in similarity scores among the two inferecne datasets, we observe a shift in sequence identity scores to functional matches in the UniProt training set.

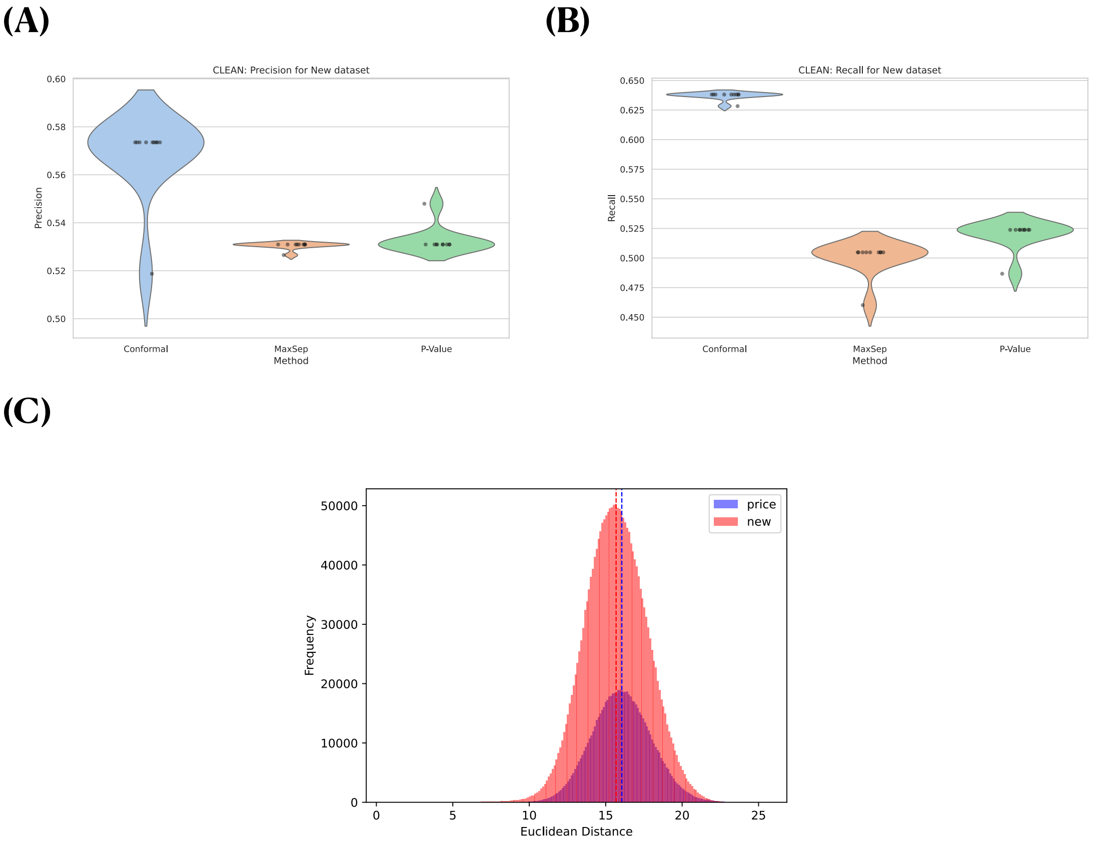

Supplementary Figure 6: **(a)** Violin plots of precision for conformal, $\mathsf{p-value}$, and $\mathsf{max-sep}$. **(b)** Violin plots of Recall for conformal, $\mathsf{p-value}$, and $\mathsf{max-sep}$. **(c)** Distribution of similarity scores in *New, Price* datasets. We observe a slight distribution shift in similarity scores between *New* and *Price*.

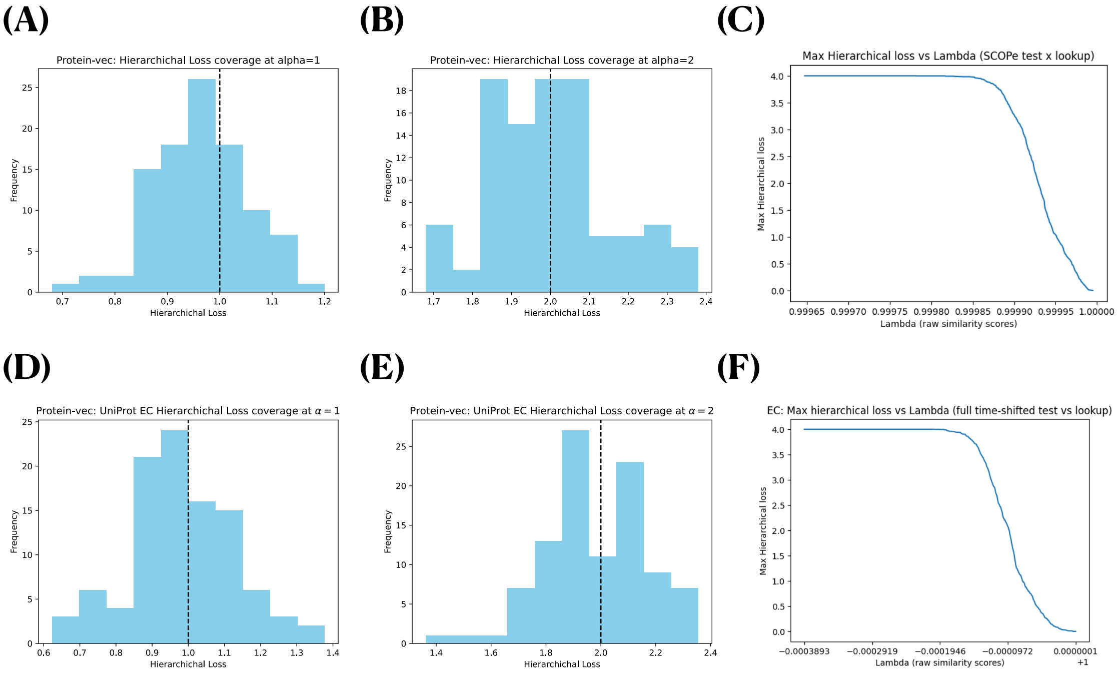

Supplementary Figure 7: **Robust calibration of hierarchical risk for SCOPe and EC searches.** We demonstrate calibration at $\alpha = 1, 2$ hierarchical losses, and report test loss over 100 trials for both. We also observe, that for both the SCOPe and EC tasks, max hierarchical loss falls as a function of similarity score threshold λ, i.e., the loss is monotone and conformal guarantees apply under conformal risk control. **(a)** SCOPe: $\alpha = 1$. $\mu(\hat{\alpha}) = 0.9645$ over 100 trials. **(b)** SCOPe: $\alpha = 2$. $\mu(\hat{\alpha}) = 1.994$ over 100 trials. **(c)** Hierarchical Loss: SCOPe. **(d)** EC: $\alpha = 1$. $\mu(\hat{\alpha}) = 0.977$ across ‘100 trials. **(e)** EC: $\alpha = 2$. $\mu(\hat{\alpha})$= 1.980 across 100 trials. **(f)** Hierarchical Loss: EC.

2 Supplementary Note 1

UniProt-wide retrieval of enzyme classes and SCOPe folds

In the previous section 3.3, we show a screening method for reducing the size of lookup databases while preserving high quality hits. Here, we instead wish to bridge the gap between conformal risk control and providing guarantees for precise classification tasks that are hierarchical in nature, such as that of protein structural and enzymatic specificity. In the process of doing so, we demonstrate how to improve interpretability for biologists searching through hierarchical data.

For instance, a common benchmark for remote homology methods is to examine model sensitivity on SCOPe, specifically the fraction of true positives (TPs) to a query protein q_i detected until the first incorrect family/fold/superfamily is detected. Similarly, as mentioned before for CLEAN, model sensitivity for EC calling is also often used to measure method performance. As a result of the hierarchical nature of these classification tasks, we aimed to establish that hierarchical and false negative rate (FNR) risk control could consistently provide coverage across many iterations of randomly shuffling the indices belonging to the held-out test set. Using an extended form of the *New* dataset, we designate 438 query proteins that pass a date cutoff from the Uniprot database used by Protein-Vec, against 211720 proteins that are filtered to have a fully-characterized EC hierarchy. We choose to do so as Aspect-Vec (a single-task Protein-Vec) model, reports better performance than CLEAN, owing to the use of per-residue embeddings over mean-pooling. Thus, we decide to use the model to simulate a harder retrieval problem than CLEAN EC-calling, where classification isn't performed simply by designating 'hits' as EC cluster centers with high similarity scores, but rather other enzymes in UniProt which may or may not even belong to an EC classification that has a set size larger than 1.

We employ two different calibration losses to infer $\hat{\lambda}$, i) the hierarchical loss and ii) false negative rate control. For both, we demonstrate, across 100 trials, that the risk is controlled across arbitrary test subsets of the SCOPe and EC tasks. For SCOPe, we measure risk control with respect to a hierarchical loss function which measures the degree of mismatch between our hierarchical prediction and the ground-truth SCOPe classification, described mathematically in the Supplement (see Eq. (7); the loss takes values in $\{0, 1, 2, 3\}$, with higher loss indicating greater mismatch). For the case of the hierarchical loss, we check for fold-level ($\alpha = 1$) and superfamily-level ($\alpha = 2$) mismatches, demonstrating that the mean loss across trials, $\hat{\alpha}$ converges to α that aligns to the constraint set during calibration (Supplementary Figure 7). We do the same for EC, measuring risk control for the same α's on the EC hierarchy, achieving similar results.
